# High-Resolution Remote Sensing Imagery Water Body Extraction Using a U-Net with Cross-Layer Multi-Scale Attention Fusion

**DOI:** 10.3390/s25185655

**Published:** 2025-09-10

**Authors:** Chunyan Huang, Mingyang Wang, Zichao Zhu, Yanling Li

**Affiliations:** School of Mathematics and Statistics, North China University of Water Resources and Electric Power, Zhengzhou 450046, China; huangchunyan@ncwu.edu.cn (C.H.); z20231080792@stu.ncwu.edu.cn (M.W.); z20231080787@stu.ncwu.edu.cn (Z.Z.)

**Keywords:** U-Net, remote sensing, water body extraction, attention mechanism

## Abstract

The accurate extraction of water bodies from remote sensing imagery is crucial for water resource monitoring and flood disaster warning. However, this task faces significant challenges due to complex land cover, large variations in water body morphology and spatial scales, and spectral similarities between water and non-water features, leading to misclassification and low accuracy. While deep learning-based methods have become a research hotspot, traditional convolutional neural networks (CNNs) struggle to represent multi-scale features and capture global water body information effectively. To enhance water feature recognition and precisely delineate water boundaries, we propose the AMU-Net model. Initially, an improved residual connection module was embedded into the U-Net backbone to enhance complex feature learning. Subsequently, a multi-scale attention mechanism was introduced, combining grouped channel attention with multi-scale convolutional strategies for lightweight yet precise segmentation. Thereafter, a dual-attention gated modulation module dynamically fusing channel and spatial attention was employed to strengthen boundary localization. Furthermore, a cross-layer geometric attention fusion module, incorporating grouped projection convolution and a triple-level geometric attention mechanism, optimizes segmentation accuracy and boundary quality. Finally, a triple-constraint loss framework synergistically optimized global classification, regional overlap, and background specificity to boost segmentation performance. Evaluated on the GID and WHDLD datasets, AMU-Net achieved remarkable IoU scores of 93.6% and 95.02%, respectively, providing an effective new solution for remote sensing water body extraction.

## 1. Introduction

Water body extraction predominantly entails the precise delineation of aquatic areas from multispectral and high-spatial-resolution remote sensing imagery. As a foundational task in remote sensing information processing, it serves a critical function in water resource management planning, ecological environment conservation, and disaster emergency response decision-making [1,2]. At present, methodologies for extracting water bodies from remote sensing imagery are primarily categorized into two types: spectral analysis techniques relying on water indices and machine learning-driven approaches.

Spectral analysis based on water indices is a commonly employed technique for remote sensing water body detection. Its core principle lies in extracting water body information by setting thresholds for reflectance differences across specific bands. The Normalized Difference Water Index (NDWI), proposed by McFeeters, serves as a foundational method for water body identification by reducing noise and shadow effects [3]. However, in complex scenarios, it is vulnerable to background interference, compromising accuracy. To address this, Xu replaced the near-infrared band in the NDWI formula with the shortwave infrared band, introducing the Modified Normalized Difference Water Index (MNDWI) [4]. This innovation significantly boosted sensitivity to water body boundaries and refined separation of water bodies from buildings and vegetation. Nevertheless, it still relies on a single threshold. To tackle misclassification issues arising from the spectral similarity between certain land features and water bodies, researchers often combine NDWI with other relevant indices. For instance, fusing the Normalized Difference Vegetation Index (NDVI) to suppress vegetation interference and incorporating the Normalized Difference Built-up Index (NDBI) to distinguish building shadows [5,6]. While such fusion strategies have achieved promising results in large-scale water body monitoring, they fundamentally fail to overcome inherent limitations, including the inability of static thresholds to adapt to the spatiotemporal variations in water body spectra, leading to unstable results, strong subjectivity in threshold selection, and poor performance in extracting small water bodies. In response to this, Szostak et al. significantly improved the extraction accuracy of micro-water bodies such as small streams by refining deep learning approaches combined with UAV data [7]; Sun et al. proposed the Combined Normalized Difference Water Index (CNDWI), which further enhanced the stability and accuracy of small water body extraction through the fusion of DEM and remote sensing images [8].

Machine learning-based water body classification methods construct feature spaces by manually designing spectral and textural features, which are then fed into classifiers. These methods circumvent the subjectivity of traditional threshold-based approaches, enhance information utilization, and demonstrate superior performance. For instance, Balázs et al. extracted water-related features from Landsat image reflectance data using principal component analysis. This approach achieved an 89% accuracy in distinguishing water bodies from saturated soils, effectively mitigating interference from soil moisture variations and successfully identifying 92% of small and medium-sized water bodies within the study area [9]. Additionally, Zhang et al.’s support vector machine (SVM) method, after optimizing kernel function parameters, reduced segmentation errors by 15.3% compared to traditional threshold-based methods in coastline extraction, with an accuracy of 90.7% for complex shorelines [10]. Nevertheless, these models commonly encounter accuracy plateaus, and their computational efficiency deteriorates significantly when handling large datasets. Manually designed features have limited descriptive power for complex water bodies, relying heavily on prior knowledge, and struggle to extract deep abstract features and global contextual information, resulting in weak model generalization and sensitivity to noise. Classifiers such as random forests, combined with hand-crafted features like scale-invariant feature transform and histogram of oriented gradients, have been applied to land surface classification, but their task-specific nature restricts cross-dataset transferability [11]. Ye et al. applied SVM to water body extraction in Dongting Lake using Landsat-8 data, achieving an overall accuracy of 91.5% with a Kappa coefficient of 0.87. The recognition accuracy for easily confused areas such as swamps and shoals was 8.3% higher than that of traditional water index methods, demonstrating the value of machine learning in water body extraction within complex wetland environments [12]. However, water index methods face challenges in threshold setting in complex terrains, and techniques like SVM and K-means are constrained by difficulties in feature engineering construction and insufficient extraction of abstract features [13,14].

The advent of deep learning has brought revolutionary breakthroughs. Deep convolutional neural networks (CNNs) automatically learn multi-level feature representations from large-scale labeled data by stacking convolutional layers, pooling layers, and batch normalization layers, circumventing the limitations of hand-crafted feature design [15,16,17]. Visual Geometry Group (VGG) network, with its concise structure of successive convolutional stacks, confirms that increasing network depth can enhance feature extraction capability, providing references for subsequent network design [18]. The fully convolutional network (FCN) first enabled end-to-end pixel-level semantic segmentation, laying the foundation for the field [19]. U-Net, with its encoder–decoder architecture and skip connections, effectively fuses shallow-level details and deep-level semantic features, significantly improving segmentation accuracy [20]. ResNet addresses the gradient problems caused by increased network depth through residual connections, achieving a synergistic optimization of depth and performance [21]. Improved models based on VGG, U-Net, and ResNet have gradually dominated water body segmentation tasks. For instance, Wang et al. employed a ResNet101 encoder with depth-wise separable convolutions combined with a multi-scale dense connection module, which significantly improved the extraction performance of small lakes in the Google remote sensing image dataset of the Tibetan Plateau. Both the overall accuracy and True Water Ratio (TWR) outperformed those of DeepLabV3+, effectively addressing the issues of large intra-class variance and small inter-class variance in lake water bodies [22]; Duan et al. introduced a multi-scale refinement network embedded with an erasure attention mechanism, which achieved excellent performance on Gaofen-1D satellite images, with high overall accuracy and MIoU. Compared to other models, it had fewer parameters, shorter training time, and improved recognition accuracy for easily confused areas [23]; Chen et al. constructed a global spatial-spectral convolution module combined with multi-scale learning, which enhanced feature extraction capability on high-resolution multispectral datasets. The Water Intersection over Union was superior to that of traditional methods, enabling effective differentiation between water bodies and building shadows [24]. Guo et al. achieved favorable extraction results for GF-1 images based on the MWEN network, with high overall accuracy. The recognition rate of small water bodies and noise suppression capability were superior to those of FCN and U-Net [25]; Peng et al. applied the improved U-Net to Zhuhai-1 hyperspectral data, achieving high Recall and Precision for small water body extraction, with accuracy superior to that of SVM and normalized water index methods [26]; Parajuli J et al. modified ResNet for Sentinel-2 images and proposed the AD-CNN model, which achieved favorable overall accuracy and MIoU on the WaterPAL dataset in Nepal, with enhanced robustness in recognizing water bodies in heterogeneous areas [27]. S. Thayammal et al. proposed a SegNet water body recognition method based on the VGG16 architecture, applied to Landsat images. By training deep hidden units to extract features, it achieved an average accuracy of 96.7%, realizing high-precision recognition performance [28]; Wang et al. developed a hybrid framework integrating Google Earth Engine’s cloud computing capabilities with multi-scale convolutional neural networks, which achieved excellent kappa coefficient and IoU on Landsat images of multiple cities, with a misclassification rate lower than that of MNDWI and random forests [29]; Li et al. validated the superiority of U-Net++ in edge extraction, water body differentiation, and non-water body suppression, with improved MIoU for edge extraction and accuracy for water/non-water body differentiation, as well as a reduced error rate for non-water body suppression [30]; Wang et al. fused the feature pyramid network with ResNet to form HA-Net, which achieved higher MIoU than MSLWENet on Google and Landsat-8 datasets, with shorter training time [31]; Kang et al. designed a multi-scale context extractor network, which achieved higher mIoU than U-Net on the LandCover.ai and DeepGlobe datasets, effectively handling water body scale variations in complex scenarios [32]. However, the local receptive field of standard convolution limits global context modeling, leading to issues such as blurred boundaries, missed detection of small tributaries, misclassification of adjacent land features, and fragmentation of linear water bodies. Consequently, the generalization ability and robustness of these models still require enhancement [33,34].

To overcome the limitations of global modeling, attention mechanisms have been introduced to enable adaptive feature focusing. Duan et al. integrated channel and spatial attention mechanisms, enhancing the continuity of water body contours [23]. Zhong et al. proposed a bidirectional channel attention mechanism, which improved the Intersection over Union for lake boundary segmentation in lake water extraction from optical remote sensing images and enhanced the ability to capture subtle boundaries [35]. However, these mechanisms still rely on convolutional operations for extracting basic features, limiting their global representation capabilities. The advent of Transformer has provided a novel paradigm for object detection tasks. By relying entirely on attention mechanisms, Transformer achieves efficient sequence modeling, serving as a substitute for conventional recurrent neural networks (RNNs) and convolutional neural networks (CNNs). Dosovitskiy et al. introduced the Vision Transformer (ViT) and applied it to image classification [36]. By dividing images into a series of uniform-sized patches and feeding them into a standard Transformer encoder, ViT achieves remarkable performance in image classification when trained with abundant computational infrastructure, such as high-performance GPU arrays. Meanwhile, the extended Long Short-Term Memory (xLSTM) network, which has emerged in recent years, has demonstrated performance comparable to that of Transformers in sequence modeling tasks through optimized gating mechanisms and enhanced memory processing capabilities, exhibiting outstanding performance in fields such as time series prediction and medical image segmentation [37,38]. Recent years have witnessed the emergence of numerous hybrid architectures that couple CNNs with Transformers. These frameworks aim to synergize CNNs’ strong local feature representation with Transformers’ superior long-range dependency modeling to develop more robust model architectures. For example, Chen et al.’s CNN–Transformer-integrated network, specialized for lake extraction, achieved high overall accuracy on multi-source remote sensing image datasets, outperforming pure CNN models and demonstrating superior segmentation performance for lakes with complex shapes [39]; Zhang et al.’s CNN–Transformer hybrid model yielded favorable overall accuracy in water body extraction from high-resolution remote sensing images, with noise suppression capability superior to traditional methods, thus reducing interference from cloud shadows and building shadows [40]; Kang et al.’s dual-stream CNN network fused with Transformer achieved high mIoU in water body detection from optical remote sensing images, outperforming single-stream CNN models and exhibiting stronger adaptability to lakes of different scales [41]. Although CNN–Transformer fusion models significantly enhance water body segmentation accuracy, Transformers require substantial computational resources to handle long-range dependencies. In contrast, CNN-based segmentation methods generally exhibit higher computational efficiency and broader practical application potential while maintaining acceptable accuracy. Therefore, research on CNN-based water body segmentation methods remains of great value.

To tackle the challenges arising from complex features and textures in remote sensing images of water bodies, this study proposes a novel U-shaped network architecture, AMU-Net, built upon the classic U-Net, and validates its performance on the GID and WHDLD datasets. The model integrates an efficient multi-scale attention mechanism, which reduces computational complexity through a grouping strategy, captures features via parallel branches, and strengthens water body features through cross-dimensional interactions and adaptive weighting, thereby enhancing adaptability across diverse scenarios. Furthermore, an improved residual connection is introduced to alleviate the vanishing gradient problem, and a gating unit is incorporated to suppress background noise. The flexible convolutional structure adapts to water bodies of varying scales, improving both training stability and segmentation accuracy.

AMU-Net’s encoder–decoder architecture integrates a dual-attention gating modulation module and a cross-layer geometric attention fusion module, which significantly boost its performance in water body segmentation tasks. The former is embedded in skip connections and employs a collaborative attention mechanism spanning spatial and channel dimensions to adaptively select and enhance key features while suppressing redundant information. The latter utilizes grouped projection convolutions and a three-level geometric attention mechanism for background suppression; through dynamic fusion and cross-layer connections, it preserves fine-grained details and strengthens scene adaptability. The synergy between these two modules enables the model to accurately capture the spatial, semantic, and geometric characteristics of the target, thereby improving recognition accuracy and localization precision in complex scenarios, enhancing scale robustness, and optimizing feature interaction and generalization capabilities. Additionally, this study proposes a triple-constraint loss framework that achieves precise segmentation by jointly optimizing global classification, regional overlap, and background specificity. In benchmark comparisons with state-of-the-art networks across multiple visual tasks, AMU-Net exhibits superior performance, validating the effectiveness and advantages of its architectural design.

The primary contributions of this study are outlined as follows:

(1) We propose the AMU-Net architecture, which integrates an efficient multi-scale attention mechanism and improved residual connections. This design effectively reduces computational complexity, mitigates the vanishing gradient problem, enhances the network’s adaptability to water bodies of varying scales, and improves both training stability and segmentation precision.
(2) We developed a dual-attention gating modulation module and a cross-layer geometric attention fusion module. Leveraging collaborative attention mechanisms across spatial, channel, and geometric dimensions, these modules enable precise selection and enhancement of water body features, thereby significantly boosting recognition accuracy and localization precision in complex scenarios.
(3) We constructed a triple-constraint loss framework that jointly optimizes three dimensions: global classification, regional overlap, and background specificity. This framework introduces a novel loss calculation paradigm for remote sensing water body segmentation tasks, facilitating more accurate segmentation results.
(4) Through comprehensive benchmarking against classic models across diverse vision tasks, we empirically demonstrate that AMU-Net exhibits superior performance in remote sensing water body segmentation, validating the efficacy of its architectural design and its competitive advantages.

The remaining sections of this paper are structured as follows: Section 2 elaborates on the proposed methodology, including the overall framework and detailed modular architectures. Section 3 describes the experimental setup, encompassing dataset specifications, cross-network comparative results, and ablation studies. Section 4 discusses the rationale behind key design choices in AMU-Net. Finally, Section 5 provides a comprehensive summary of the research and highlights its contributions.

## 2. Proposed Method

### 2.1. General Architectural Framework of AMU-Net

**Figure 1:**
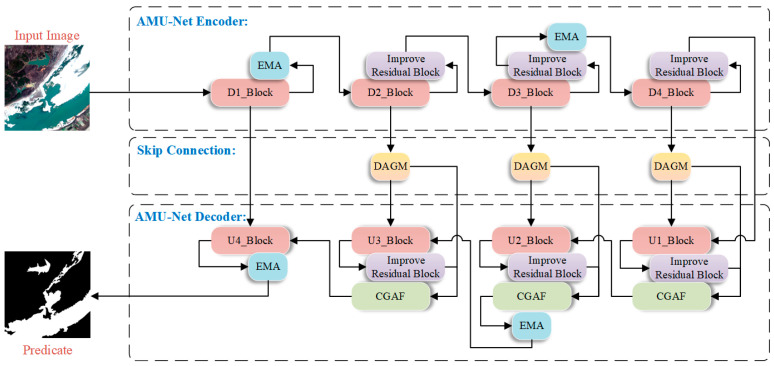
Schematic diagram of the AMU-Net architecture.

The AMU-Net model is constructed on the U-Net framework, achieving multi-scale feature decoupling, joint geometric-semantic modeling, and computational efficiency optimization through hierarchical multi-module embedding and cross-layer attention mechanisms. Employing a classic encoder-decoder architecture, the model consists of four-level downsampling modules, four-level upsampling modules, and improved skip connections, as illustrated in Figure 1.

AMU-Net’s encoder comprises modules D1_Block through D4_Block. Each incorporates two successive ReLU-activated 3 × 3 convolutional layers and a 2 × 2 max pooling layer for downsampling. The quantity of feature channels is doubled at each downsampling step until the minimum resolution is attained. After D1_Block completes the basic feature extraction process, an Efficient Multi-scale Attention (EMA) module is integrated to enhance the discrimination between water bodies and backgrounds through cross-spatial interactions and multi-branch attention mechanisms. This preserves spatial-semantic information in high-resolution features and mitigates water edge blurring caused by downsampling. Similarly, after downsampling in D3_Block, the EMA module is introduced to calibrate channel weights using global information, capturing contextual correlations of large-scale water bodies in remote sensing images and improving the model’s semantic understanding of complex scenes.

In the skip connections of AMU-Net, shallow features are optimized by adding Dual Attention Gating Modulation (DAGM) modules at the outputs of D2–D4 layers. These modules use attention mechanisms to filter high-response features related to water bodies and suppress irrelevant background information, ensuring that features transmitted to the decoder are more targeted and addressing the information mixing issue in traditional skip connections.

In AMU-Net’s decoder, modules U1_Block through U4_Block retain abundant feature channels during upsampling to transmit context toward higher-resolution layers. Each employs a 2 × 2 transposed convolution that halves channel dimensionality, followed by fusion with corresponding encoder features, dual 3 × 3 convolutional layers, and ReLU activation. Additionally, U2_Block and U4_Block incorporate extra EMA modules to alleviate information loss during upsampling, enabling more accurate restoration of water contours—particularly in small water bodies or fragmented regions—to enhance spatial consistency of features and improve edge clarity and overall segmentation accuracy. The Cross-Scale Guided Attention Fusion (CGAF) module is innovatively introduced in the U1–U3 layers of the decoder to enhance interaction and integration of features across different levels. By fusing upsampled features with high-resolution features from corresponding encoder layers, the CGAF module effectively integrates multi-scale contextual information and spatial details.

AMU-Net incorporates enhanced residual blocks across its architecture. These units adjust feature map values by embedding sigmoid activation within residual connections. This scales inputs to [0, 1], controlling information flow through enhancing key features while suppressing irrelevant ones. The modified connections retain the primary advantage of residual blocks—improving gradient flow via skip connections—and condition inputs for more stable, efficient gradient propagation.

### 2.2. Improved Residual Connection Module

In the field of high-resolution remote sensing image analysis, accurately capturing the spatial details of complex geographic features—such as small-scale water bodies—remains a critical challenge in water body extraction tasks. While the classic U-Net architecture has demonstrated excellent performance in semantic segmentation, it exhibits inherent limitations in representing features within complex aquatic environments. Remote sensing datasets contain numerous tiny water bodies, which are highly intertwined with heterogeneous land cover types, posing stringent requirements on the model’s ability to discriminate fine details. Although the downsampling path of U-Net can effectively capture global contextual information, the aggregation of multi-scale features inevitably leads to the attenuation of high-frequency details. This significantly degrades the model’s accuracy in delineating the boundaries of complex shapes, such as narrow streams and fragmented water bodies.

To address the above issues, this study embeds residual connection modules into the U-Net architecture to construct a multi-scale feature enhancement mechanism for preserving high-resolution detail information. As a core component of the ResNet architecture, residual connections enable the direct interaction of features across different layers through explicit modeling of cross-layer identity mapping paths [42].

In the improved AMU-Net model (Figure 1), each convolutional block achieves element-wise fusion of input features and non-linearly transformed outputs through skip connections, with this operation performed before the activation function to ensure that the network’s learning objective shifts from complete feature transformation to residual mapping. This optimization enables the model to focus on incremental feature learning of high-frequency details, significantly enhancing its ability to characterize complex water body shapes. Experimental results show that the U-Net with residual blocks achieves a significant improvement in water body boundary localization accuracy compared to the traditional U-Net, particularly with high-resolution remote sensing imagery, demonstrating notably superior pixel-level classification accuracy.

**Figure 2:**
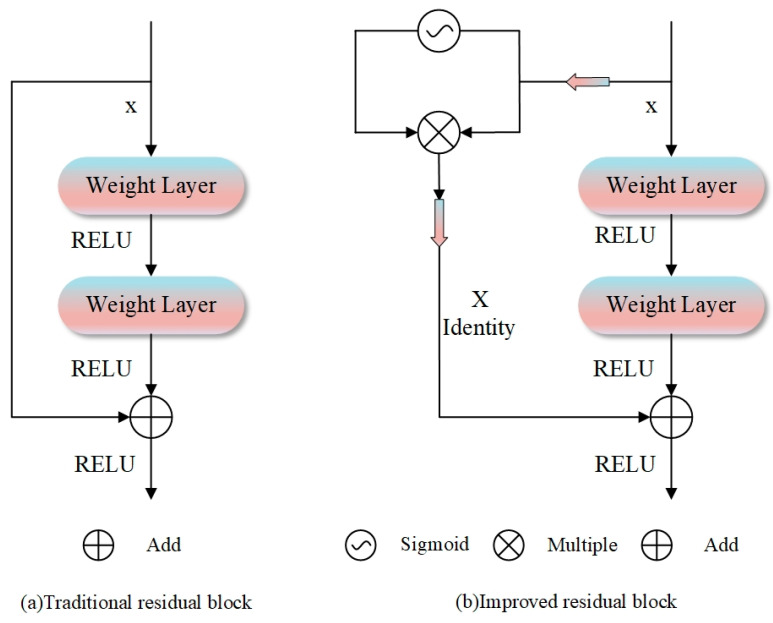
Two residual block architectures: (**a**) traditional design with a direct skip connection for residual learning; (**b**) improved version with a sigmoid-gated mechanism.

To further optimize the structure of residual connections, a dynamic feature regulation method based on a gating mechanism is proposed, as illustrated in Figure 2. By introducing adaptive weights controlled by a Sigmoid activation function in skip connections, the proposed method dynamically optimizes the information balance between the identity pathway and the transformed pathway. The mechanism enables the fine-grained modulation of cross-layer information flow through element-wise multiplication, enhancing the model’s adaptability to multimodal input data while effectively mitigating overfitting. By introducing dynamic regulation parameters, the gated residual block maintains training stability and significantly reduces the risk of gradient anomalies in deep networks, providing a new technical paradigm for precise water body extraction in complex remote sensing scenarios.

### 2.3. Efficient Multi-Scale Attention Module

Although the improved residual block mitigates information loss via cross-layer connections and markedly boosts feature propagation efficiency in water body extraction models, it does not explicitly model the importance of feature channels. For water body extraction in remote sensing images, features from different spectral channels and spatial locations play distinct roles in water body semantics. Residual connections only offer pathways for information flow and cannot dynamically adjust each channel’s response intensity, potentially diluting key water body features with background noise and undermining the model’s discriminative capacity in complex scenarios.

Traditional channel attention mechanisms can enhance key features by modeling cross-channel dependencies, yet they often rely on channel dimensionality reduction, which may introduce information loss in deep features. Additionally, single-scale attention modeling struggles to adapt to the multi-scale nature of water body targets in remote sensing images and lacks the fine-grained capability to capture spatial pixel-level semantic correlations. To address these issues, we introduce the EMA mechanism, an attention mechanism combining channel grouping and cross-spatial interactions [43]. It captures global context and local feature relationships through parallel branches, enabling lightweight multi-scale feature enhancement.

**Figure 3:**
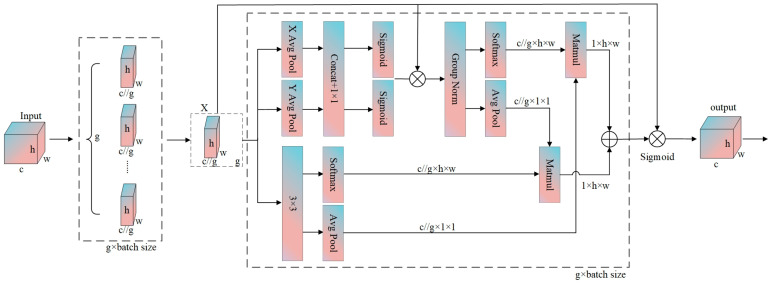
Structural diagram of an efficient multi-scale attention mechanism.

The structural diagram of the EMA mechanism is shown in Figure 3. Through multi-scale channel grouping and lightweight modeling, it divides channels into several subgroups and reshapes them into the batch dimension, reducing computational complexity while balancing the distribution of spatial semantic features within subgroups to adapt to the multi-scale characteristics of water bodies. It captures cross-spatial interactions and pixel-level correlations via horizontal and vertical adaptive pooling and 1×1 convolutions, strengthening the fine-grained semantic discrimination of water body boundaries. Feature fusion is achieved through cross-dimensional interactions of parallel branches for global context and local details, aggregating multi-scale features to avoid one-sided modeling.

Specifically, for an input feature map $X\in R^{B\times C\times H\times W}$, the EMA module first partitions the channels into G groups, yielding

(1) $$Group_{X}\in R^{B}\times G\times \frac{C}{G}\times H\times W$$

Spatial context is extracted via horizontal pooling $X_{h}=AvgPool_{H\left(Group_{X}\right)}$ and vertical pooling $X_{w}=AvgPool_{W\left(Group_{X}\right)}^{⊤}.$ After fusion by 1 × 1 convolution, spatial attention weights are generated:

(2) $$M_{spatial}=\sigma \left({Conv}_{1\times 1}\left(\left[X_{h},X_{w}\right]\right)\right)$$

These weights modulate the grouped features:

(3) $$F_{1}=GroupNorm\;\left(Group_{X}\cdot M_{spatial}\right)$$

Meanwhile, local features are extracted via 3 × 3 convolution:

(4) $$F_{2}={Conv}_{3\times 3}\left({Group}_{X}\right)$$

Global average pooling is applied to $F_{1}$ and $F_{2}$, followed by Softmax to generate channel attention weights $A_{1}$ and $A_{2}$. Cross-spatial dependency is calculated via matrix multiplication:

(5) $$W=\sigma \left(\begin{array}{c} A_{1}\cdot F_{2}^{\mathrm{flat}}+A_{2}\cdot F_{1}^{\mathrm{flat}} \end{array}\right)$$

where $F_{1}^{flat}$ and $F_{2}^{flat}$ denote flattened features. Finally, the grouped features are fused with attention weights and reshaped to produce the output:

(6) $$Y=Reshape\;\left(Group_{X}\cdot W,B\times C\times H\times W\right)$$

This module reduces computational load through grouping, enhances multi-scale features via spatial and channel attention, and improves the model’s spatial–semantic capture capability under lightweight design.

In the baseline U-Net model, the encoder extracts multi-scale features from shallow to deep levels through progressive downsampling of the original image. Shallow features primarily capture details such as water body edges and textures, while deep features focus on semantic-level representations of overall water body shapes and distribution patterns. The decoder then fuses deep semantic features with shallow detail features via upsampling and skip connections, ultimately enabling pixel-level classification predictions. In this process, the model’s segmentation accuracy for water body targets depends heavily on the expressive power of deep features and the effectiveness of feature fusion.

Considering the diverse scales of water bodies and complex backgrounds in remote sensing images, this study deploys the EMA mechanism at the first and third downsampling layers of the encoder, as well as the second and fourth upsampling layers of the decoder. At the first downsampling layer of the encoder, the EMA extracts features with different receptive fields in parallel using multi-branch dilated convolutions, enabling it to simultaneously capture the detailed course of rivers and the contour structures of small lakes at medium resolutions. This effectively prevents feature blurring caused by single-scale convolutions. At the third downsampling layer, the EMA enhances semantic understanding of large-scale watershed distributions during deep semantic encoding by dynamically fusing multi-scale information. It also preserves the detailed features of small targets such as scattered ponds and mitigates information loss during downsampling.

In the decoder, the EMA at the second upsampling layer—acting after cross-level feature fusion—suppresses background noise from land vegetation and buildings through joint channel and spatial attention weighting, strengthening the semantic alignment of boundaries between water and non-water bodies. The EMA at the fourth upsampling layer addresses the need for detail restoration during upsampling by refining texture information (e.g., ripples on the water surface and water shorelines) through multi-scale feature analysis. It also ensures consistency between detailed expressions and high-level semantics, thereby significantly improving the segmentation accuracy of small water bodies and water body edges in complex environments.

### 2.4. Dual-Attention-Gated Modulation Module

**Figure 4:**
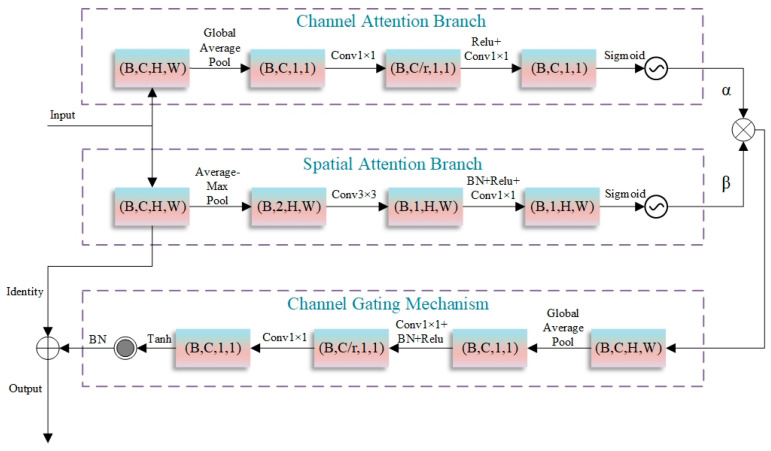
Structure of the dual-attention-gated modulation module.

In the context of semantic segmentation for high-resolution remote sensing images, shallow features contain abundant spatial detail information but often lack high-level semantic information and are vulnerable to noise interference. Although traditional attention mechanisms can enhance feature representation to some extent, they fail to fully address the problem of feature optimization in complex scenarios of remote sensing images. To this end, this paper proposes a novel DAGM to refine features. By fusing channel attention and spatial attention, and introducing a learnable weight balancing mechanism, the module further enhances the model’s capability to identify targets like water bodies. As depicted in Figure 4, the DAGM adaptively optimizes input features through the parallel processing of channel and spatial attention, along with the incorporation of a gating mechanism and residual connections.

The DAGM consists of several key components: a dual-channel attention mechanism, an adaptive attention weight balancing module, a channel gating mechanism, and residual connections. In the dual-channel attention mechanism, the channel attention branch captures global statistical feature information via global average pooling, compresses and reconstructs the channel dimension using two 1×1 convolutions, and generates channel attention weights with a sigmoid function. The channel compression ratio r is set to 16 and adjustable according to task complexity. The spatial attention branch extracts spatial features by combining average and max pooling, enhances spatial information representation via 3 × 3 depthwise separable convolutions, and generates spatial attention weights through 1 × 1 convolutions. The adaptive attention weight balancing module introduces learnable parameters $channel_{weight}$ and $spatial_{weight}$, which are normalized by the sigmoid function to dynamically adjust the contribution ratio of the two attentions, enabling the model to adaptively allocate attention resources based on input features. The channel gating mechanism re-modulates attention-enhanced features, generating gating weights through global information perception. The tanh activation function confines weights to [−1, 1], allowing for the selective enhancement or suppression of features. The residual connection module preserves original feature information, helping mitigate the vanishing gradient problem in deep network training.

Specifically, for an input feature map $X\in R^{B\times C\times H\times W}$, the DAGM module first calculates channel attention:

(7) $$M_{c}\left(X\right)=\sigma \left(W_{2}\cdot \mathrm{ReLU}\left(W_{1}\cdot \mathrm{GAP}\left(X\right)\right)\right)$$

where GAP denotes global average pooling, $W_{1}$ and $W_{2}$ are 1 × 1 convolution operations, and σ is the sigmoid activation function. Simultaneously, spatial attention is computed as

(8) $$M_{s}\left(X\right)=\sigma \left({\mathrm{Conv}}_{1\times 1}\left(\mathrm{ReLU}\left(\mathrm{BN}\left({\mathrm{Conv}}_{3\times 3}\left(\left[\mathrm{AvgPool}\left(X\right);\mathrm{MaxPool}\left(X\right)\right]\right)\right)\right)\right)\right)$$

where $\left[\cdot ;\cdot \right]$ represents feature concatenation, and $C{\mathrm{onv}}_{k\times k}\;$ denotes a k×k convolution. Learnable parameters are introduced as

$$\begin{array}{c} \alpha =\sigma \left(channel_{weight}\right)\\ \beta =\sigma \left(spatial_{weight}\right) \end{array}$$

where α and β are learnable attention balancing parameters. The influence of the two attentions on the original features is fused to obtain

(9) $$\hat{X}=X\cdot \left(\alpha \cdot M_{c}\left(X\right)\right)\cdot \left(\beta \cdot M_{s}\left(X\right)\right)$$

Furthermore, a feature-modulating coefficient is learned through the gating mechanism:

(10) $$Gate\left(\hat{X}\right)=Tanh\left(W_{4}\cdot ReLU\left(BN\left(W_{3}\cdot GAP\left(\hat{X}\right)\right)\right)\right)$$

where $W_{3}$ and $W_{4}$ are 1 × 1 convolution operations, and tanh is the hyperbolic tangent activation function. Subsequently, channel gating is applied: $Y=\hat{X}\cdot \left(1+Gate\left(\hat{X}\right)\right)$, and the final output is generated via residual connection: $Output=BN\left(Y\right)+X.$

The DAGM module is designed by integrating a collaborative attention mechanism across spatial and channel dimensions, an adaptive weight balancing strategy, an information retention mechanism, and a lightweight architecture. These advantages enable it to effectively suppress interference from non-water bodies in water body recognition tasks, thereby improving segmentation accuracy and model robustness. The design characteristics of DAGM underscore its potential to tackle the complexities of remote sensing image analysis in intricate scenarios, presenting an innovative technical pathway to improve the accuracy of water body information extraction.

### 2.5. Cross-Layer Geometry–Attention Fusion

In remote sensing water body segmentation tasks, high-spatial-resolution satellite imagery displays substantial variations in multi-scale water body morphology, texture, and contextual cues, which are frequently confused with backgrounds like vegetation shadows and urban structures, presenting critical challenges to segmentation accuracy. Traditional Convolutional Neural Networks (CNNs), limited by their single-scale feature representation capabilities, struggle to effectively handle such complex scenes. Additionally, the inherent problem of CNNs—shallow features rich in spatial details but weak in semantics, and deep features strong in semantic abstraction but losing spatial information—further exacerbates the difficulty of precise multi-scale water body segmentation. Therefore, designing cross-layer feature fusion mechanisms to integrate complementary information across different levels has become critical to improving segmentation accuracy.

**Figure 5:**
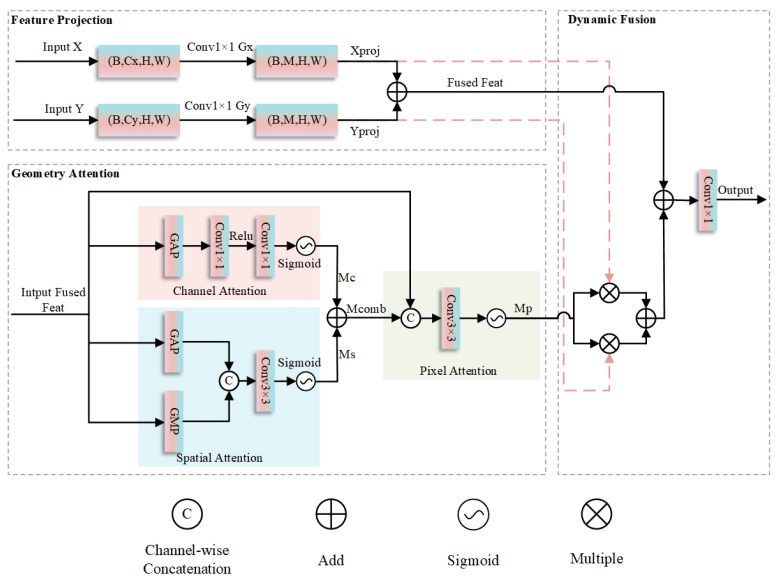
Structure diagram of Cross-Layer Geometric–Attention Fusion.

To tackle the aforementioned challenges, this research introduces a CGAF module, as illustrated in Figure 5, which achieves adaptive integration of cross-layer features and precise modeling of water body semantics through the collaborative interaction of spatial, channel, and pixel three-dimensional attention mechanisms. The module first maps feature maps from different layers to the same intermediate dimension via 1 × 1 convolutions. After initial fusion through element-wise summation, multi-level geometric attention mechanisms are introduced sequentially. The spatial attention sub-module first performs average and max pooling on the fused features, concatenates the pooling results, and applies a 3 × 3 convolution to generate a spatial attention map, thereby enhancing spatial key regions such as water body boundaries. The channel attention sub-module then applies global average pooling to the fused features, learns channel dependencies via a multi-layer perceptron composed of two 1 × 1 convolutions, and generates a channel attention map to enhance channel responses corresponding to water body spectral features. The pixel attention sub-module subsequently concatenates the fused features with the first two attention maps, generates pixel-level attention weights through a 3 × 3 convolution and sigmoid activation, and achieves fine-grained feature calibration. Finally, a weighted fusion strategy is employed to dynamically enhance or suppress input feature branches using pixel attention weights; multi-dimensional enhanced features are integrated via residual connections, and channel mapping is completed through 1 × 1 convolutions to output the final result.

Specifically, for input feature maps $X\in R^{B\times C_{x}\times H\times W}$ and $Y\in R^{B\times C_{y}\times H\times W}$, the CGAF module first projects X and Y into an intermediate dimension $C_{\mathrm{mid}}$ via grouped convolutions:

(11) $$X_{\mathrm{proj}}={\mathrm{GroupConv}}_{1\times 1,G_{x}}\left(X\right),\;Y_{\mathrm{proj}}={\mathrm{GroupConv}}_{1\times 1,G_{y}}\left(Y\right)$$

where $G_{x}=max\left(1,C_{x}/64\right)$ and $G_{y}=max\left(1,C_{y}/64\right)$ are the number of groups, satisfying $C_{\mathrm{mid}}\%G_{x}=0$ and $C_{\mathrm{mid}}\%G_{y}=0$. Initial fused features are obtained via element-wise addition:

(12) $$F_{\mathrm{fused}}=X_{\mathrm{proj}}+Y_{\mathrm{proj}}$$

Next, channel attention for $F_{\mathrm{fused}}$ is calculated as

(13) $$M_{c}\left(F_{\mathrm{fused}}\right)=\sigma \left({\mathrm{Conv}}_{1\times 1}\left(\mathrm{ReLU}\left({\mathrm{Conv}}_{1\times 1}\left(\mathrm{GAP}\left(F_{\mathrm{fused}}\right)\right)\right)\right)\right)$$

where GAP denotes global average pooling, and the two convolutional layers have channel dimensions $C_{\mathrm{mid}}\to C_{\mathrm{mid}}/r\to C_{\mathrm{mid}}$ (r is the compression ratio). Spatial attention for $F_{\mathrm{fused}}$ is computed as

(14) $$M_{s}\left(F_{\mathrm{fused}}\right)=\sigma \left({\mathrm{Conv}}_{3\times 3,\mathrm{pad}=1}\left(\left[\mathrm{AvgPool}\left(F_{\mathrm{fused}}\right);\mathrm{MaxPool}\left(F_{\mathrm{fused}}\right)\right]\right)\right)$$

where $\left[;\right]$ represents channel concatenation and the convolution uses reflection padding. The combined attention map is $M_{\mathrm{comb}}=M_{c}\left(F_{\mathrm{fused}}\right)+M_{s}\left(F_{\mathrm{fused}}\right)$. A pixel attention module then generates pixel-level attention maps by concatenating $F_{\mathrm{fused}}$ and $M_{\mathrm{comb}}$ followed by convolution:

(15) $$M_{p}=\sigma \left({\mathrm{Conv}}_{3\times 3,\mathrm{pad}=1}\left(\left[F_{\mathrm{fused}};M_{\mathrm{comb}}\right]\right)\right)$$

Finally, features are fused via residual-weighted aggregation:

(16) $$F_{\mathrm{enhanced}}=F_{\mathrm{fused}}+M_{p}\cdot X_{\mathrm{proj}}+\left(1-M_{p}\right)\cdot Y_{\mathrm{proj}}$$

The output is generated via a 1×1 convolution:

(17) $$\mathrm{Output}={\mathrm{Conv}}_{1\times 1}\left(F_{\mathrm{enhanced}}\right)$$

Here, σ is the sigmoid activation function. Grouped convolutions and concatenation operations are used to reduce computational load and fuse multi-dimensional features, respectively.

In remote sensing water body segmentation tasks, the CGAF module demonstrates notable advantages. For large-scale water bodies, the module captures their holistic shapes and contextual correlations via high-level semantic features, while refining boundaries using low-level detail features, thus mitigating confusion with backgrounds such as shadows. For small-scale water bodies, it leverages fine-grained spatial information from low-level features for precise localization, addressing the issue of small-target information loss caused by downsampling in traditional methods. Furthermore, through its multi-level attention mechanisms, the module effectively suppresses interference from irrelevant backgrounds and enhances the model’s robustness against environmental factors like illumination changes and seasonal variations. By efficiently integrating cross-layer feature information, the CGAF module provides a more comprehensive and accurate feature representation for remote sensing water body segmentation, exhibiting strong practicality and promotional value.

### 2.6. Joint Loss Function

Water body segmentation in high-spatial-resolution remote sensing imagery faces challenges: difficulty extracting small-target features due to class imbalance, discontinuous segmentation results from fuzzy water–land boundaries, and false positive detections caused by spectrally similar ground objects in complex backgrounds. Traditional single-loss functions have notable limitations here: loss gradients are easily dominated by the large-proportion background class, leading to insufficient learning of small-target features; pixel-level classification strategies ignore the structural consistency of target regions, making it hard to maintain spatial continuity of segmentation results; moreover, such loss functions lack semantic discrimination mechanisms for spectrally similar ground objects, failing to address semantic confusion from feature overlap and ultimately limiting improvements in water body segmentation accuracy.

To address these challenges, this paper proposes a triple-constraint loss framework to achieve precise segmentation by collaboratively optimizing global classification, regional overlap, and background specificity. The total loss function is defined as

(18) $$L_{Total}=L_{CE}+L_{Dice}+L_{BCE-BG}$$

The cross-entropy loss $L_{CE}$ maximizes the log-likelihood of class posterior probabilities, forcing the model ta learn discriminative features in spectrally confused scenarios [44]. Its mathematical expression is

(19) $$L_{CE}=-\frac{1}{N}\sum_{i=1}^{N}\sum_{c=1}^{C}y_{i,c}log\left({\hat{y}}_{i,c}\right)$$

where N is the total number of pixels, $y_{i,c}$ is the one-hot-encoded ground truth label, and ${\hat{y}}_{i,c}=softmax\left(f_{i,c}\right)$ is the predicted probability. Derived from probability theory, this loss function stabilizes the optimization of classifier outputs, effectively distinguishing spectrally similar backgrounds from water bodies, and provides fundamental classification capabilities for segmentation tasks. The Dice loss tackles class imbalance arising from the minimal proportion of water bodies in remote sensing images by optimizing the pixel-wise overlap between prediction results and ground truth masks [45]. Its mathematical formulation is

(20) $$L_{Dice}=1-\frac{2\sum_{i,c}y_{i,c}{\hat{y}}_{i,c}}{\sum_{i,c}y_{i,c}+\sum_{i,c}{\hat{y}}_{i,c}}$$

In binary classification scenarios, it simplifies to

(21) $$L_{Dice}=1-\frac{2\left|A\cap B\right|}{\left|A\right|+\left|B\right|}$$

where A and B represent the ground truth and predicted water regions, respectively. By directly acting on regional overlap, this loss function enables the model to focus on the integrity of small targets, significantly improving the continuity and accuracy of segmentation boundaries. The $L_{BCE-BG}$ (background binary cross-entropy) loss is designed for spectrally similar objects in remote sensing backgrounds, such as wet soil and shoals. Its formula is

(22) $$L_{BCE-BG}=-\frac{1}{N}\sum_{i=1}^{N}\left[y_{i,0}log\left(\sigma \left(f_{i,0}\right)\right)+\left(1-y_{i,0}\right)log\left(1-\sigma \left(f_{i,0}\right)\right)\right]$$

where $\sigma \left(\cdot \right)$ is the sigmoid function, and $f_{i,0}$ is the raw output for the background class. This loss enhances the model’s semantic understanding of complex backgrounds by independently constraining background class prediction probabilities, reducing false detections in water–land transition zones, and improving scene adaptability.

The hybrid loss function capitalizes on the distinct advantages of each individual loss component to effectively boost water body segmentation performance in high-spatial-resolution remote sensing imagery. The integration of the three losses not only ensures the strictness of classification logic but also enhances adaptability to the intrinsic challenges of remote sensing data, thereby significantly elevating the overall accuracy and reliability of water body segmentation results.

## 3. Experimental

To fully validate the effectiveness of the proposed model, this study conducts a series of comparative experiments and ablation tests. In the comparative experiments, the model and methods are compared with several current popular semantic segmentation models; the ablation tests focus on analyzing the roles of each component of the model to comprehensively evaluate its performance.

### 3.1. Experimental Datasets

The datasets used in this study are the GID dataset and the WHDLD dataset. Both are publicly available datasets widely applied in numerous fields.

#### 3.1.1. GID Dataset

The GID Dataset [46] is sourced from high-resolution remote sensing imagery acquired by China’s Gaofen-2 satellite. As a pivotal element of the National Space Administration’s High-Resolution Earth Observation System, the Gaofen-2 satellite is outfitted with dual panchromatic and multispectral (PMS) sensors. Its panchromatic images boast a spatial resolution of 1 m, whereas the multispectral ones attain a 4-m resolution, with a single scan spanning a 45-km-wide swath. The post-processing of the satellite-derived data enhances the panchromatic image resolution in the dataset to 0.8 m and the multispectral one to 3.24 m, while keeping each camera’s viewing angle at 2.1 degrees. The dataset comprises 150 images accompanied by pixel-level annotations. The multispectral bands cover blue (0.45–0.52 μm), green (0.52–0.59 μm), red (0.63–0.69 μm), and near-infrared (0.77–0.89 μm) ranges. Each image has dimensions of 6800 × 7200 pixels, exhibiting abundant ground object texture details.

To tackle the limitations of the original GID dataset and align with the research aims of this paper, the GID dataset underwent the subsequent processing procedures.

**Figure 6:**
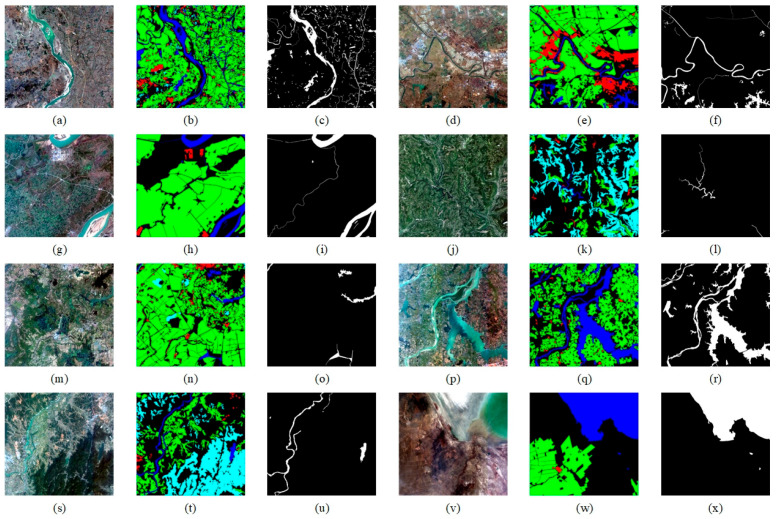
The GID dataset and its labels. (**a**,**d**,**g**,**j**,**m**,**p**,**s**,**v**) are original images. (**b**,**e**,**h**,**k**,**n**,**q**,**t**,**w**) are multi-category label images. (**c**,**f**,**i**,**l**,**o**,**r**,**u**,**x**) are label images with only water and non-water bodies.

(a) Considering the high-resolution characteristics of the original dataset images, which could lead to excessive memory usage and low computational efficiency during training, this paper employs a block cropping technique to uniformly crop the original remote sensing images and their corresponding label maps into 512 × 512-pixel sub-images.
(b) To resolve the issue of unbalanced sample category distribution (e.g., significant differences in the number of water and non-water samples shown in Figure 6g–i,m–o), the dataset was filtered by removing all image samples without water bodies. This significantly increased the proportion of water samples in the overall dataset.
(c) Given the complexity of annotation work for water body extraction tasks, the original six categories were simplified into two classes: water and non-water.
(d) To safeguard the scientific validity of model training and evaluation, the processed dataset was partitioned into training and validation subsets following an 8:2 ratio.

As shown in the labeling system of the GID dataset (Figure 6), it uses a six-class label division: buildings are annotated in red, farmland in green, forests in light blue, grasslands in yellow, water bodies in dark blue, and unclassified areas in black. While this dataset furnishes ample data for remote sensing multi-task classification, it exhibits constraints in water body recognition applications. On one hand, the massive amount of non-water images and complex labeling system increase data processing complexity; on the other hand, the dataset does not explicitly partition training and validation sets, which poses certain obstacles to model training and performance evaluation.

#### 3.1.2. WHDLD Dataset

**Figure 7:**
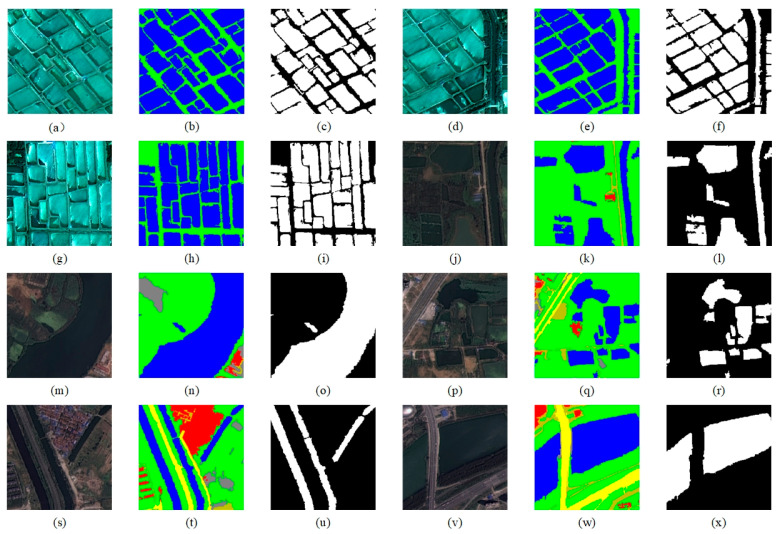
The WHDLD dataset and its labels. (**a**,**d**,**g**,**j**,**m**,**p**,**s**,**v**) are original images. (**b**,**e**,**h**,**k**,**n**,**q**,**t**,**w**) are multi-category label images. (**c**,**f**,**i**,**l**,**o**,**r**,**u**,**x**) are label images with only water and non-water bodies.

The WHDLD dataset [47], released by Wuhan University in 2018, comprises 4940 high-resolution remote sensing images with a 2-m resolution. Each image, measuring 256 × 256 pixels and having an 8-bit depth, consists of three channels (conventional RGB images). This dataset encompasses six types of remote sensing ground objects, such as buildings, vegetation, and water bodies (Figure 7). To center on the core research question of this paper, the original data labels were initially binarized and re-categorized into two classes: water and non-water. Afterward, the structured dataset was divided into training and validation sets following an 8:2 ratio. In the model training stage, data augmentation techniques were employed on the training set, involving random flipping and rotation operations.

### 3.2. Experimental Environment Configuration and Evaluation Metrics

In this experiment, the hardware platform was equipped with four Tesla V100 PCIE 16 GB LS graphics cards, providing a total video memory capacity of 64 GB. The operating system used was Linux, and the software environment was built on Python 3.9.21. The deep learning framework adopted was PyTorch 2.5.1, integrated with the CUDA 12.1 acceleration component. To thoroughly assess the model’s efficacy in the water body extraction task, validation was performed at the end of each training epoch. Six pixel-level metrics—mean accuracy, precision, intersection over union, recall, F1 score, and boundary F1 score—were used for comprehensive evaluation of the model’s performance.

Specifically, average accuracy (Equation (23)) refers to the arithmetic mean of per-class correct prediction proportions, assessing the model’s multi-category classification capability. Precision measures the fraction of actual water pixels among predicted water pixels, reflecting prediction reliability. IoU is the ratio of overlap area between predicted and ground-truth annotations. Recall measures the percentage of correctly identified water pixels among all actual water pixels, assessing positive sample capture capability. The F1 score (Equation (27)) combines precision and recall via their harmonic mean, avoiding over-optimization toward either metric. Boundary F1 Score is calculated the same way as F1 score but focuses on water boundary pixels, evaluating edge contour prediction accuracy. Formulas follow

(23) $$Average\;Accuracy=\frac{1}{N}\sum_{i=1}^{N}\frac{TP_{i}+TN_{i}}{TotalSamples_{i}}$$

(24) $$Precision=\frac{TP}{TP+FP}$$

(25) $$IoU=\frac{TP}{TP+FP+FN}$$

(26) $$Recall=\frac{TP}{TP+FN}$$

(27) $$F1\;Score=2\cdot \frac{Precision\cdot Recall}{Precision+Recall}$$

where $TP_{i}$ stands for true positive, representing the count of pixels accurately predicted as water bodies within class i; $TN_{i}$ denotes true negative, signifying the count of pixels correctly predicted as non-water bodies in class i; $TotalSamples_{i}$ is the overall pixel count in class i; FP is false positive, referring to the quantity of non-water body pixels misidentified as water bodies; FN is false negative, indicating the quantity of water body pixels misclassified as non-water bodies. In the calculation of the boundary F1 score, the statistical scope of the above parameters is limited to the pixels in the water boundary area.

IoU is sensitive to water boundary precision and enables the evaluation of contour depiction capability; the F1 score balances precision and recall, enhancing small water body detection and the complete extraction of large water areas; the boundary F1 score focuses on edge details, supplementing the assessment of boundary coincidence in segmentation results. All metrics are based on confusion matrices, ensuring objective comparability.

### 3.3. Effectiveness Analysis of the Joint Loss Function

To validate the effectiveness of the proposed triple-constraint loss, this study conducted loss function ablation experiments on the baseline U-Net model, where the weights of individual losses in the joint loss function were all set to 1. Table 1 presents how different loss functions perform in water body extraction tasks. Comparative experiments for each loss function combination were independently repeated 10 times, and the final metrics were derived by averaging the results across these repetitions to mitigate random errors. Experimental results indicate that the proposed triple-constraint loss function attains the optimal performance in water body segmentation tasks across both the GID and WHDLD datasets.

Specifically, on the GID dataset, Dice loss alone alleviates class imbalance by optimizing regional overlap but lacks classification and background-specific constraints, resulting in low average accuracy of 0.9705, precision of 0.9594, a boundary F1 score of only 0.7436, susceptibility to interference from spectrally similar objects, and proneness to blurred or discontinuous segmentation boundaries; introducing cross-entropy loss enhances the model’s discriminative ability, with average accuracy increasing to 0.9712, precision to 0.9608, and boundary F1 score to 0.7614, effectively distinguishing water from confusing classes like shadows and soil and improving boundary localization; adding BCE-BG loss further boosts semantic discrimination for complex backgrounds such as wet soil and shoals, with average accuracy reaching 0.9728, IoU 0.9117, precision 0.96, and boundary F1 score 0.7775, reducing false detections and enhancing boundary continuity and clarity. The triple-loss fusion, via synergistic global classification constraints, regional overlap optimization, and background suppression, comprehensively resolves class imbalance, boundary blurring, and background misdetection, validating the necessity of multi-loss complementarity for improving water segmentation accuracy, and both the baseline U-Net and all modified models adopt this triple-constraint loss to ensure consistent validity in subsequent experiments.

### 3.4. Comparison of Water Body Extraction Accuracy with Existing Models

To systematically verify the effectiveness and superiority of AMU-Net, this study carried out comparative experiments against classic semantic segmentation models, including DeepLabV3+ [48], FCN [49], SegNet [50], UNet++ [51], U2-Net [52], HA-Net [31], MAFUNet [53], rkformer [54], and dim2clear [55]. For each model involved in the comparison, 10 independent training and testing processes were performed, and the reported metrics are the average of the 10 results. Table 2 and Table 3 present the performance of diverse network architectures in water body extraction tasks on the GID and WHDLD datasets.

Experimental results show that the baseline U-Net model exhibits relatively limited performance across all evaluation metrics. In contrast, AMU-Net achieves significant performance improvements through its four integrated innovative modules. Specifically, on the GID dataset, AMU-Net achieves an average accuracy of 0.9801, an IoU of 0.9360, a recall of 0.9654, an F1-score of 0.9668, and a boundary F1-score of 0.8283, with all metrics significantly surpassing the baseline model. Although DeepLabV3+ performs comparably to AMU-Net in certain metrics, its boundary segmentation accuracy is significantly lower, with suboptimal delineation of water edges in complex remote sensing scenarios; MAFUNet shows strong overall performance, closely trailing AMU-Net and demonstrating impressive capability in capturing boundary details, placing it among the top performers in boundary segmentation. UNet++ and U2-Net show strong performance in accuracy and recall, with UNet++ ranking among the leading models in boundary segmentation highlighting its effectiveness in capturing boundary information in specific scenarios whereas U2-Net demonstrates moderate boundary processing capability. HA-Net achieves solid performance across basic metrics but lags in boundary segmentation, reflecting limitations in handling fine edge details. dim2clear achieves excellent overall performance with top-tier boundary segmentation, reflecting its effectiveness in handling water edge details; in contrast, rkformer lags in both overall accuracy and boundary processing, indicating limitations in adapting to complex remote sensing water scenarios. Notably, while SegNet and FCN show some comparability to AMU-Net in basic metrics, their boundary segmentation accuracy remains consistently low, and in complex water scenarios involving noise, shadows, or scale variations, the segmentation boundaries generated by these models lack integrity and clarity. U-Net, despite maintaining decent overall performance, exhibits moderate boundary processing capability, falling short of the top performers in capturing fine water edges. Overall, experimental results confirm that AMU-Net achieves superior performance in remote sensing water segmentation, particularly in boundary segmentation, and additionally, due to their lightweight nature, FCN and SegNet serve as viable solutions for resource-constrained scenarios, fully validating the scientific rationale and effectiveness of the AMU-Net architecture.

**Figure 8:**
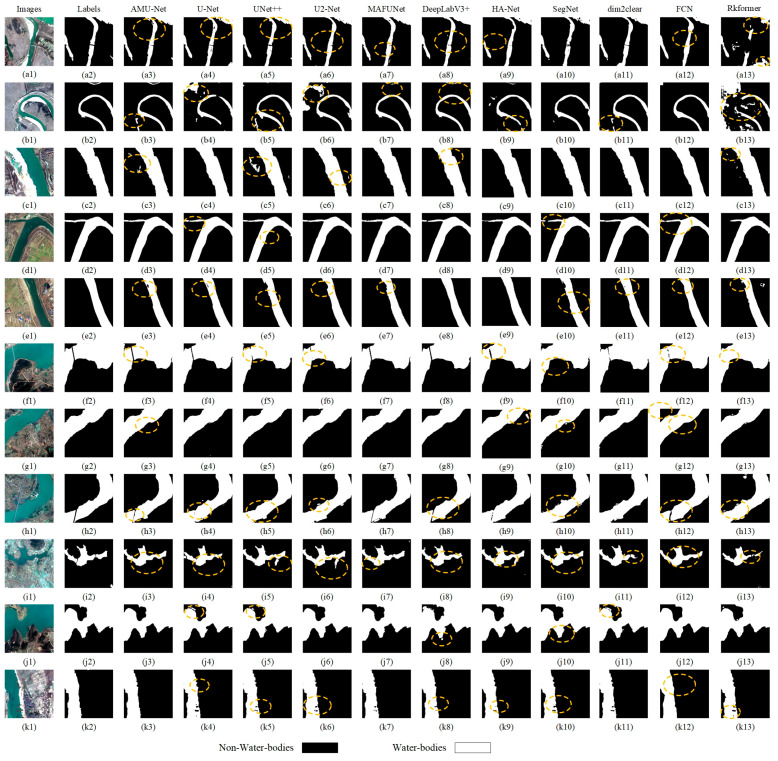
Evaluates multiple semantic segmentation models on the GID dataset, including AMU-Net, U-Net, U-Net++, U2-Net, MAFUnet, DeepLabV3+, HA-Net, SegNet, dim2clear, Rkformer, and FCN. It uses original images (**a1**–**k1**), with corresponding labels (**a2**–**k2**): black regions mark non-water bodies and white regions indicate water bodies, (**a3**–**k3**) to (**a13**–**k13**) correspond to the prediction result maps of the aforementioned 11 semantic segmentation models (the model corresponding to each group of prediction maps should be specified in the figure or its accompanying legend, e.g., (**a3**–**k3**) for AMU-Net, (**a4**–**k4**) for U-Net, etc.).For the circled areas in the subfigures: The circled regions are located in the prediction result maps (**a3**–**k3**) to (**a13**–**k13**) and are highlighted to emphasize key performance characteristics of the models.

This subsection presents a comparative analysis of water body segmentation results across networks, illustrated in Figure 8. In Figure 8’s first row (a1), AMU-Net, MAFUNet, and dim2clear can capture finer water areas, which are highly consistent with the ground truth (a2); FCN, U2-Net, and HA-Net exhibit omissions in key regions; SegNet and other networks have classification errors at the edges and for small water bodies; and rkformer (a13) performs the worst with severe distortion. In (b1), AMU-Net, UNet++, and dim2clear show excellent boundary classification and can identify subtle unlabeled water bodies, among which AMU-Net performs better at key positions; DeepLabV3+ is slightly inferior with weaker boundary processing capability; and other networks have distortion and misclassification, with rkformer (b13) being the worst. As image complexity increases, rows (c) and (d) show differences in the models’ handling of complex backgrounds. AMU-Net and UNet++ perform comparably, with AMU-Net being superior at key positions. In Figure 8(e1), AMU-Net, UNet++, MAFUNet, and dim2clear are better in terms of continuity and integrity when detecting small aquatic obstacles. When bridges cross water bodies, as shown in Figure 8(f1), SegNet is prone to misclassification, UNet++ and others achieve partial recognition, while AMU-Net, MAFUNet, and DeepLabV3+ have higher segmentation accuracy, with AMU-Net being more precise at key positions, and rkformer showing severe misclassification. Overall, AMU-Net, MAFUNet, and dim2clear are better at preserving the geometric shape and boundary clarity of water bodies, with AMU-Net being superior at key positions, DeepLabV3+ being slightly inferior, and rkformer being the worst.

Further prediction results demonstrate the performance of each network in different water body scenarios. In Figure 8(g1), most models can identify large water bodies, while rkformer, SegNet, and HA-Net misclassify non-water areas, with AMU-Net being better at key positions. In Figure 8(h1), AMU-Net, MAFUNet, and DeepLabV3+ are superior to others in recognizing pier structures, with DeepLabV3+ and MAFUNet being slightly inferior in identifying key positions. In Figure 8(i1,j1), AMU-Net and the other three networks show prominent capabilities in maintaining the shape and boundary integrity of complex water bodies, with AMU-Net performing better at key positions. With increased scene complexity Figure 8(k1), AMU-Net’s ability to restore the boundary integrity of water bodies surpasses others, but it has difficulty in identifying obstacles in water bodies. These results indicate that AMU-Net, UNet++, MAFUNet, and dim2clear have stronger accuracy and robustness in processing small or complex water bodies, and their boundary processing is among the top performers, with AMU-Net being superior at key positions, DeepLabV3+ being slightly inferior, and rkformer being the worst.

To substantiate the superiority of the AMU-Net framework in water body extraction, this study implemented it on the WHDLD dataset, with the results presented in Table 3. Compared with the metrics in Table 2, all models exhibit significant improvements in their performance metrics. This phenomenon can be attributed to the relatively homogeneous scene types and fewer interfering factors in the WHDLD dataset, which facilitate the models in learning stable water body features. Among these models, AMU-Net still ranks first in all metrics, while dim2clear, MAFUNet, and HA-Net perform slightly inferior to AMU-Net. The water body extraction performance of other models shows no significant difference compared with that on the GID dataset. In conclusion, the AMU-Net model achieves the optimal performance in water body extraction tasks.

Table 4 presents a comparison of model complexity among different networks with an input size of 512 × 512. In terms of model complexity, AMU-Net has 29.66 MB parameters, 225.17 G FLOPs, 0.47 GB inference memory consumption, and 0.59 GB training memory consumption. Compared with models of similar performance, its parameter scale and computational load are higher than those of UNet++, MAFUNet, and U-Net, and its inference and training memory requirements are also higher than these lightweight models. Although DeepLabv3+ has lower FLOPs, AMU-Net exhibits superior comprehensive performance. This indicates that while maintaining high performance, AMU-Net has relatively high complexity, leaving room for further lightweight optimization.

Future optimizations can focus on reducing model complexity while maintaining high-precision segmentation capabilities. For example, exploring more efficient feature fusion modules to reduce parameter redundancy, or introducing dynamic network mechanisms to adaptively adjust computing resources, thereby improving the applicability of the model in resource-constrained scenarios while preserving its advantages in segmenting complex water boundaries.

### 3.5. Ablation Experiment Study

**Figure 9:**
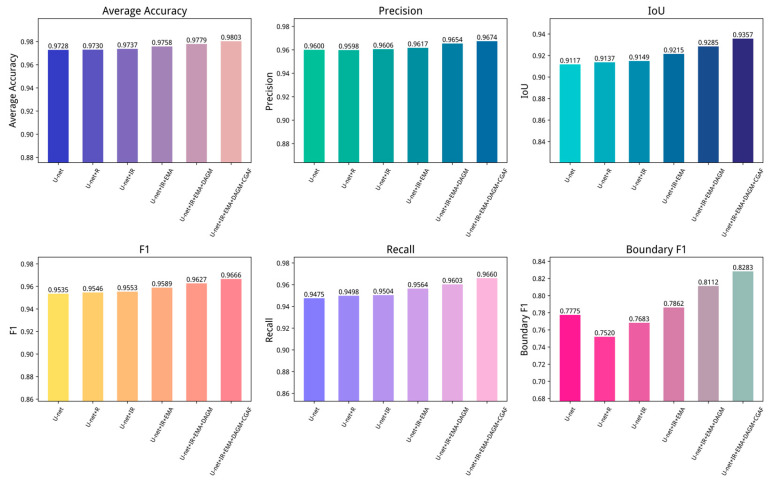
Ablation experiments on the GID dataset. R represents the traditional residual block, IR represents the improved residual block, EMA represents the efficient multi-scale attention module, DAGM represents the dual-attention gating modulation, and CGAF represents the cross-layer geometric attention fusion module.

**Figure 10:**
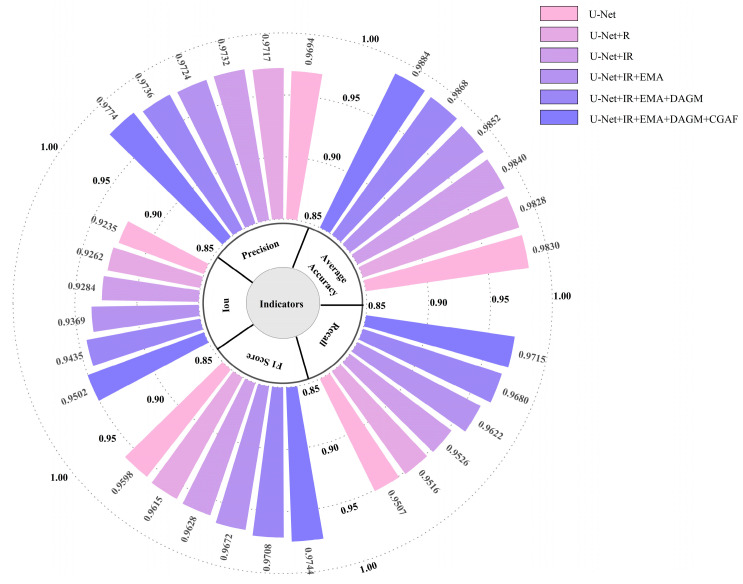
Ablation experiments on the WHDLD dataset. R represents the traditional residual block, IR represents the improved residual block, EMA represents the efficient multi-scale attention module, DAGM represents the dual-attention gating modulation, and CGAF represents the cross-layer geometric attention fusion module.

To evaluate the individual impact of different modules on water body segmentation tasks, ablation experiments were conducted by progressively introducing various model components into the baseline U-Net model. Each ablation group was run 10 times, and the average of the 10 results was used to evaluate the impact of different components. To clearly demonstrate experimental accuracy, the performance of each trial was summarized on the GID and WHDLD datasets, with the results presented in Table 5 and Table 6. Further visualizations of network test results are shown in Figure 9 and Figure 10.

**Figure 11:**
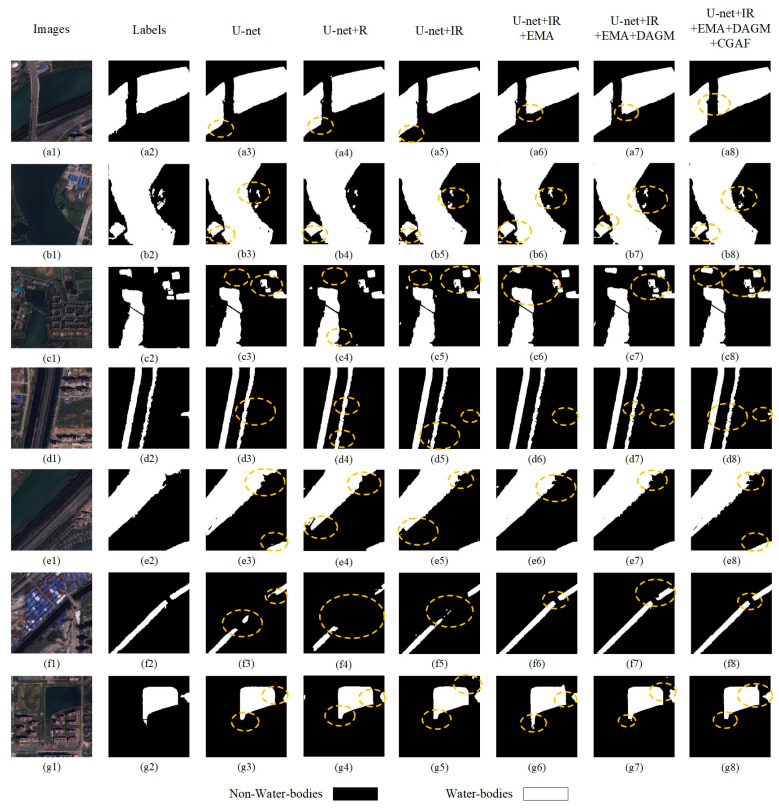
U-Net comparisons with the added modules. (**a1**–**g1**) depict original images; (**a2**–**g2**) are ground truth labels (black indicates non-water bodies, white represents water bodies); (**a3**–**g3**) to (**a8**–**g8**) represent the prediction result maps of the baseline U-Net model with different modules added. For the circled areas in the subfigures: The circled regions are located in the prediction result maps (**a3**–**g3**) to (**a8**–**g8**) and are highlighted to emphasize key performance characteristics of the models.

To visually demonstrate the impact of various modules on water body segmentation, this paper presents recognition results in Figure 11. The baseline U-Net exhibits preliminary segmentation capability but suffers from relatively high average loss, indicating significant potential for enhancement. As noted in Figure 11(b3,c3,f3), U-Net loses substantial semantic information in ambiguous scenes and misclassifies complex water bodies. Introducing R residual blocks dramatically drops the model’s average loss and improves metrics including accuracy, precision, IoU, and F1-score, demonstrating their role in capturing deep features and enhancing learning capacity. However, as observed in Figure 11(c4,e4), R blocks alone yield suboptimal water extraction outcomes, despite the network beginning to form basic contour understanding. After integrating improved residual blocks, as shown in Figure 9 and Figure 10, most metrics of the network improve. Combining the fourth and fifth columns of Figure 11, the network’s boundary prediction capability is notably enhanced, verifying the effectiveness of the improved residual blocks.

Additionally, incorporating the EMA mechanism, the U-Net + IR + EMA model shows gains in all performance metrics, suggesting that the attention mechanism enhances segmentation accuracy by sharpening attention to salient features. U-Net + IR + EMA effectively classifies the image in Figure 11(c1), achieves segmentation accuracy for complex-texture water bodies in (b6) and (c6), and identifies the tiny river in (f6) with greater continuity and completeness. Finally, the DAGM and CGAF modules enable optimal performance, reflected in notable IoU and F1-score gains. This indicates multi-scale feature fusion improves handling of diverse-sized water bodies, crucial for model enhancement. Finally, introducing the DAGM and CGAF modules allows the model to reach optimal performance, notably seen in IoU and F1-score improvements. This implies fusing features across scales helps the model better manage water bodies of varying sizes, critical for enhancing model performance.

As observed in prediction images, the final network finely categorizes the complex texture in Figure 11(c1) and performs well across all predicted images-especially in handling water bodies with complex terrain and blurred boundaries, where AMU-Net shows superior adaptability and robustness. For instance, in regions with highlights and shadows, AMU-Net accurately distinguishes water from non-water regions. These results confirm that each component enhances performance, especially in capturing fine details.

## 4. Discussion

As satellite technology advances, research on extracting water bodies from high-resolution remote sensing imagery has gained increasing focus. The complexity of water bodies in shape, size, and other dimensions makes extracting them highly challenging [56]. For example, reflected light in high-altitude regions and ambiguous ground features can easily misclassify water bodies. Since 2014, deep learning has increasingly become the dominant method for target classification in remote sensing imagery, so leveraging it to extract water bodies from high-resolution data holds substantial development potential. This study introduces the AMU-Net model, an enhancement of the U-Net architecture. Using five performance metrics and practical predictions, AMU-Net demonstrates superior ability to extract water bodies with complex textures compared to other networks.

The performance disparities among different semantic segmentation models in water body extraction tasks stem from their unique architectural designs and feature processing mechanisms. As a classic baseline model, U-Net relies on a “U”-shaped symmetric structure, extracting features through convolutional pooling in the contraction path and reconstructing images via upsampling and cross-layer feature fusion in the expansion path, enabling effective segmentation even in data-limited scenarios. Building upon this, UNet++ constructs a multi-level feature fusion network through densely nested skip connections, effectively alleviating the semantic gap between features in traditional U-Net and significantly improving water body boundary segmentation accuracy. U2-Net employs a dual U-shaped nested architecture combined with deep supervision to achieve efficient interaction of multi-scale features, and is capable of simultaneously capturing the global contours and local texture details of water bodies, making it particularly suitable for complex texture scenarios. As a lightweight model, SegNet retains boundary information while reducing parameter counts through upsampling strategies that use max-pooling indices in the decoder, maintaining high segmentation efficiency in resource-constrained environments. dim2clear enhances edge detail preservation through adaptive feature refinement, and shows strong robustness in handling water bodies with uneven illumination. Rkformer integrates transformer and convolutional mechanisms via random-connection attention, strengthening the capture of small-scale water body features. MAFUNet adopts multi-scale attention fusion to balance global context and local texture, improving segmentation consistency for irregular water bodies. HA-Net leverages hybrid attention mechanisms to enhance discriminability between water and spectrally similar features, and performs well in large-scale high-resolution imagery. As a pioneer of fully convolutional networks, FCN achieves end-to-end pixel-level classification yet lacks accuracy stability when dealing with water bodies of varying sizes, as it lacks a multi-scale feature fusion mechanism. DeepLabV3+ strengthens the capture of multi-scale contextual information of water bodies through dilated convolutions and spatial pyramid pooling modules; its decoder design further improves boundary segmentation accuracy, and is especially suitable for complex urban environments.

Extracting water bodies from high-resolution remote sensing images confronts multi-dimensional technical hurdles. First, complex boundaries limit segmentation accuracy-when water bodies adjoin vegetation, shadows, or other ground objects, boundary geometry fuzziness rises, heightening precise segmentation difficulty. Second, spectral confusion is prominent: the similarity in spectral features between water bodies and non-water regions like wetlands and shadows easily causes model misclassification. Third, the diversity of water body morphology, scale, and environmental conditions exacerbates recognition complexity: detecting small-scale water bodies imposes higher requirements on network sensitivity. Fourth, variations in water body spectral features caused by lighting changes and shadow interference further increase recognition difficulties. Fifth, high-resolution images have large data volumes and high information redundancy, posing severe challenges to data processing capabilities, storage space, and computational resources. These technical bottlenecks urgently require breakthroughs through optimized algorithm design, innovative network architectures, and improved computational paradigms to achieve high-precision and efficient water body extraction.

Two datasets were used in the experiments. The first is the GID dataset, containing over 8780 labeled training images of 512 × 512 pixels and over 2195 validation images of the same size and labeling. The second dataset, WHDLD, includes 4000 labeled 256 × 256-pixel training images and 1000 same-sized, similarly labeled validation images. Experiments ran on an A100 graphics card. For the GID dataset, the baseline U-Net model needed 8.5 h to train 100 epochs with a batch size of 32. Under these conditions, AMU-Net took 12.3 h, while DeepLabV3+ needed 9.7 h. Training durations for U2-Net, UNet++, dim2clear, and rkformer fell between 9–10 h. MAFUNet and HA-Net needed around 8.5–9 h of training under the same setup, while lightweight networks FCN and SegNet finished training in under 8 h. On the WHDLD dataset, all networks finished training in under 8 h. Though AMU-Net has marginally higher computational costs, it remains the optimal choice for accuracy and water body segmentation performance.

## 5. Conclusions

Accurately extracting water bodies from high-resolution remote sensing imagery remains a challenging task that requires models with refined feature recognition capabilities. This study proposes AMU-Net, an enhanced model based on the U-Net architecture, specifically designed for Gaofen-2 satellite imagery. By deeply integrating advanced deep learning techniques, AMU-Net significantly improves the accuracy of water body extraction.

Experimental results demonstrate that AMU-Net outperforms eight mainstream CNN-based semantic segmentation models across multiple benchmark tests. The model exhibits excellent performance on all evaluation metrics, particularly demonstrating exceptional generalization capability in complex environments. Ablation studies confirm the critical role of its core modules in handling multi-scale water bodies and easily confusable features. In typical scenarios such as urban rivers and plateau lakes, AMU-Net accurately segments water targets of various morphologies while effectively suppressing non-aqueous interference, confirming its strong robustness.

With further dataset optimization and architectural refinement, AMU-Net shows strong potential to become a core tool for high-resolution water body mapping, providing reliable technical support for dynamic water resource monitoring and management. This study not only broadens the technical approaches for water extraction, but also offers valuable references for subsequent research in remote sensing image semantic segmentation.

However, this study has several limitations. The extraction capability for small water bodies such as narrow streams and ditches, as well as artificial structures within water bodies, requires further improvement. The spatiotemporal variations of water bodies caused by seasonal, climatic, and human factors impose higher demands on model stability. Additionally, the relatively high computational complexity of the model limits its deployment in resource-constrained environments. Future work will focus on enhancing small target detection, improving spatiotemporal adaptability, and exploring lightweight designs to optimize practical application efficiency.

## Figures and Tables

**Table 1:** Ablation experiments on the loss function of the baseline model U-Net on GID and WHDLD datasets. Significant values are in bold.

Data	GID			WHDLD		
Loss Function	Dice	Dice + CE	Dice + CE + BCE-BG	Dice	Dice + CE	Dice + CE + BCE-BG
Average Accuracy	0.9705	0.9712	**0.9728**	0.9808	0.9821	**0.9830**
Precision	0.9594	0.9608	**0.9600**	0.9596	0.9680	**0.9694**
IoU	0.9062	0.9081	**0.9117**	0.9188	0.9204	**0.9235**
F1 Score	0.9505	0.9516	**0.9535**	0.9575	0.9582	**0.9598**
Recall	0.9421	0.9429	**0.9475**	0.9554	0.9489	**0.9507**
Boundary F1 score	0.7436	0.7614	**0.7775**	0.7460	0.7659	**0.7804**

**Table 2:** Accuracy comparison with other networks on the GID dataset. Significant values are in bold.

Method	Average Accuracy	Precision	IoU	F1	Recall	Boundary F1
AMU-Net	**0.9801**	**0.9683**	**0.9360**	**0.9668**	**0.9654**	**0.8283**
dim2clear	0.9756	0.9647	0.9214	0.9589	0.9535	0.8135
rkformer	0.9605	0.9520	0.8750	0.9328	0.9149	0.7015
MAFUNet	0.9754	0.9670	0.9212	0.9588	0.9510	0.8245
HA-Net	0.9694	0.9529	0.9031	0.9487	0.9449	0.7675
UNet++	0.9766	0.9628	0.9241	0.9603	0.9581	0.8012
U2-Net	0.9738	0.9612	0.9168	0.9563	0.9518	0.7727
SegNet	0.9711	0.9631	0.9072	0.9510	0.9397	0.6958
FCN	0.9715	0.9610	0.9088	0.9520	0.9435	0.7068
DeepLabv3+	0.9790	0.9713	0.9320	0.9646	0.9583	0.7712
U-Net	0.9728	0.9600	0.9117	0.9535	0.9475	0.7775

**Table 3:** Accuracy comparison with other networks on the WHDLD dataset. Significant values are in bold.

Method	Average Accuracy	Precision	IoU	F1	Recall	Boundary F1
AMU-Net	**0.9884**	**0.9774**	**0.9502**	**0.9744**	**0.9715**	**0.8702**
dim2clear	0.9881	0.9760	0.9492	0.9738	0.9717	0.8658
rkformer	0.9856	0.9754	0.9382	0.9680	0.9607	0.8478
MAFUNet	0.9869	0.9772	0.9436	0.9708	0.9647	0.8678
HA-Net	0.9829	0.9706	0.9272	0.9621	0.9538	0.7783
UNet++	0.9877	0.9767	0.9471	0.9727	0.9688	0.8671
U2-Net	0.9867	0.9759	0.9427	0.9703	0.9649	0.8191
SegNet	0.9872	0.9748	0.9452	0.9717	0.9687	0.7911
FCN	0.9745	0.9513	0.8945	0.9441	0.9371	0.6503
DeepLabv3+	0.9864	0.9769	0.9417	0.9699	0.9631	0.7219
U-Net	0.9830	0.9694	0.9235	0.9598	0.9507	0.7804

**Table 4:** Model complexity comparison of different networks (512 × 512 input).

Model	Params (MB)	FLOPs (G)	Inf. Mem. (GB)	Train. Mem. (GB)
AMU-Net	29.66	225.17	0.47	0.59
dim2clear	20.36	229.38	0.35	0.45
rkformer	29.75	98.41	0.48	0.61
MAFUNet	17.35	160.84	0.29	0.37
HA-Net	34.33	83.84	0.50	0.64
UNet++	9.16	139.62	0.17	0.21
U2-Net	44.16	159.93	0.72	0.92
SegNet	29.44	160.68	0.48	0.60
FCN	19.38	205.00	0.32	0.40
DeepLabv3+	40.94	46.97	0.65	0.81
U-Net	17.26	160.76	0.29	0.36

**Table 5:** Ablation experiments on WHDLD, Significant values are in bold.

Method	Average Accuracy	Precision	IoU	F1	Recall	Boundary F1
U-net	0.9830	0.9694	0.9235	0.9598	0.9507	0.7804
U-net + R	0.9828	0.9717	0.9262	0.9615	0.9516	0.7676
U-net + IR	0.9840	0.9732	0.9284	0.9628	0.9526	0.7719
U-net + IR + EMA	0.9852	0.9724	0.9369	0.9672	0.9622	0.8040
U-net + IR + DAGM + AMM	0.9868	0.9736	0.9435	0.9708	0.9680	0.8313
U-net + IR + EMA + DAGM + CGAP	**0.9884**	**0.9774**	**0.9502**	**0.9744**	**0.9715**	**0.8702**

**Table 6:** Ablation experiments on GID, Significant values are in bold.

Method	Average Accuracy	Precision	IoU	F1	Recall	Boundary F1
U-net	0.9728	0.9600	0.9117	0.9535	0.9475	0.7775
U-net + R	0.9730	0.9598	0.9137	0.9546	0.9498	0.7520
U-net + IR	0.9737	0.9606	0.9149	0.9553	0.9504	0.7683
U-net + IR + EMA	0.9758	0.9617	0.9215	0.9589	0.9564	0.7862
U-net + IR + DAGM + AMM	0.9779	0.9654	0.9285	0.9627	0.9603	0.8112
U-net + IR + EMA + DAGM + CGAP	**0.9803**	**0.9674**	**0.9357**	**0.9666**	**0.9660**	**0.8283**

## Data Availability

The data supporting the findings of this study are available in Baidu AI Studio Dataset at the following URLs: Wuhan High-Resolution Remote Sensing Image Dataset (WHDLD, reference number 103120) and GID-5 Dataset (reference number 153467), accessed on 23 February 2025. The GID-5 Dataset is originally sourced from https://x-ytong.github.io/project/GID.html (accessed on 28 February 2025), and both datasets are hosted on Baidu AI Studio.
